# Nursing and the Climate Crisis - The Call of COP30 for Health in Latin America

**DOI:** 10.1590/1518-8345.0000.4680

**Published:** 2025-06-02

**Authors:** Ethel Leonor Maciel

**Affiliations:** 1 Universidade Federal do Espírito Santo, Vitória, ES, Brazil.

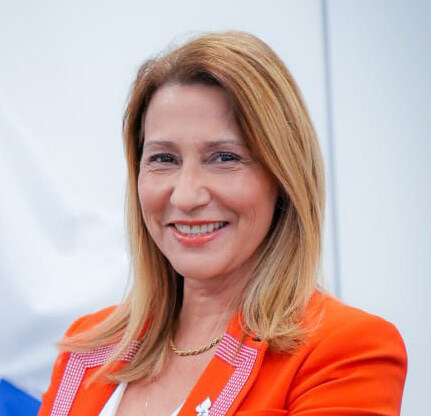



The climate emergency is one of the greatest threats to global health in the 21st century. The declaration approved during the G20 ministerial meeting in Brazil, titled “Ministerial Declaration on Climate Change, Health, Equity, and One Health”, solidifies this milestone by recognizing the climate crisis as a direct threat to global health, especially for vulnerable populations. The document emphasizes the need to integrate climate and health policies to combat zoonotic diseases and the effects of extreme events such as heatwaves, floods, and droughts. G20 leaders also committed to prioritizing the adaptation of health systems, ensuring equitable access to resources and technologies, and increasing funding for projects that reduce greenhouse gas emissions in the health sector^(1)^.

In Latin America, where the effects of global warming intertwine with historical inequalities, nursing emerges not only as a witness but also as a protagonist in building ethical and effective responses. As global leaders prepare for the 30th Conference of the Parties to the United Nations Framework Convention on Climate Change (COP30), to be held in November 2025 in Belém, Pará, Brazil, an urgent question arises: how can we transform human care into collective action for the planet?

The region presents a striking paradox: despite harboring nearly 40% of global biodiversity, it is among the most vulnerable to climate change impacts. Over the past decade, Brazil has seen a 35% increase in flooding events, while the incidence of tropical diseases like dengue and chikungunya has doubled. Moreover, Indigenous communities - responsible for conserving roughly 80% of the remaining forests - have faced rising rates of child malnutrition and malaria outbreaks directly linked to deforestation^(2)^.

Nursing, which represents 56% of healthcare professionals in the region^(3)^, experiences these crises in three dimensions: a) Healthcare delivery: dehydrated children from Amazonian heatwaves, elderly patients with pneumonia worsened by urban air pollution; b) Epidemiological: surveillance of leptospirosis outbreaks post-floods, mapping arbovirus risk zones, and rising malaria cases; c) Political: lack of sanitation in urban peripheries, where pediatric hospitalizations are tied to waterborne diarrheal diseases, and absent policies to curb air pollution - responsible for annual premature deaths - overburdening already fragile health systems.

The choice of the Amazon as COP30’s host is no coincidence. It reflects both the imminent crisis of a biome critical to global climate regulation and the historical resistance of traditional peoples, whose ancestral knowledge safeguards critical ecosystems. For nursing, this conference presents three imperatives:

1. Equitable Financing

Despite climate issues directly impacting health, only 0.5% of global climate mitigation and adaptation funds are allocated to healthcare. Yet, the economic toll of environmentally linked diseases in Latin America exceeds $2 billion annually^(4)^. Redirecting funds is essential for:

• Disaster-resilient hospital infrastructure.

• Continuous environmental health training for nurses, with clear targets by 2030.

2. Science Integrated with Traditional Knowledge

Conventional protocols often fail in contexts like riverine or Indigenous land-management practices (e.g., agroforestry systems, which reduce vector-borne diseases by up to 40%^(2)^). Nursing must bridge Western science and traditional wisdom to shape culturally appropriate, sustainable policies.

3. Sustainable and Resilient Health Systems

The health sector itself contributes 4.4% of global CO₂ emissions^(5)^. Adopting renewables (e.g., solar energy in hospitals) and reducing plastic waste are urgent steps. While global examples exist, Latin America lags behind. Such measures boost both environmental sustainability and institutional resilience^(6)^.

COP30 is not just another conference. It is a chance for Latin American nursing to lead a movement uniting science, policy, and social justice. Key steps include:

• Integrating climate crisis into nursing curricula to prepare professionals for environmental uncertainty.

• Strengthening interdisciplinary research on climate impacts on maternal-child, mental, and occupational health.

• Securing decision-making roles - from local councils to COP30 delegations - to reject neutrality in the face of devastation.

The climate crisis demands that nursing transcend its traditional role and champion a future where caring for people and the planet are two sides of the same coin. History will judge us by our courage to address not just symptoms but also the root injustices scarring bodies and lands. Inaction is not an option. Forged in struggle and resistance, Latin American nursing must rise once more to declare: there is no true care without justice, and no future without planetary health.
