# Supplementary material for: Nursing and the Climate Crisis - The Call of COP30 for Health in Latin America
Source: Rev Lat Am Enfermagem. 2025 Jun 2;33:e4680. doi: 10.1590/1518-8345.0000.4680 (PMC12129445; doi:10.1590/1518-8345.0000.4680)
Supplement: Translation

# Translation: Enfermagem e a crise climática - o chamado da COP30 para a saúde na América Latina

**DOI:** 10.1590/1518-8345.0000.4681

**Authors:** Ethel Leonor Maciel

**Affiliations:** 1 Universidade Federal do Espírito Santo, Vitória, ES, Brasil.



A emergência climática é uma das maiores ameaças à saúde global do século XXI. A declaração aprovada durante a reunião ministerial do G20 no Brasil, intitulada “Declaração Ministerial sobre Mudança Climática, Saúde e Equidade e Uma Só Saúde”, consolida esse marco ao reconhecer a crise climática como uma ameaça direta à saúde global, especialmente para populações em situação de vulnerabilidade. O documento reforça a necessidade de integrar políticas climáticas e sanitárias para combater doenças zoonóticas e os efeitos de eventos extremos, como ondas de calor, enchentes e secas. Os líderes do G20 também se comprometeram a priorizar a adaptação dos sistemas de saúde, garantindo acesso equitativo a recursos e tecnologias, e a ampliar o financiamento para projetos que reduzam a emissão de gases de efeito estufa no setor saúde^(1)^.

Na América Latina, onde os efeitos do aquecimento global se entrelaçam com desigualdades históricas, a enfermagem emerge não apenas como testemunha, mas como protagonista na construção de respostas éticas e eficazes. Enquanto líderes globais se preparam para a 30ª Conferência das Partes da Organização das Nações Unidas sobre Mudanças Climáticas (COP30), a ser sediada em novembro de 2025 em Belém-Pará, Brasil, urge refletir: como transformar o cuidado humano em ação coletiva pelo planeta?

A região apresenta um paradoxo marcante: apesar de abrigar cerca de 40% da biodiversidade global, figura entre as mais vulneráveis aos efeitos das mudanças climáticas. Nos últimos dez anos, observou-se um incremento de 35% na frequência de eventos de inundação no Brasil, ao passo que a incidência de doenças tropicais, como dengue e chikungunya, duplicou. Ademais, as comunidades indígenas - responsáveis pela conservação de aproximadamente 80% das florestas remanescentes - têm sido acometidas por elevados índices de desnutrição infantil e por surtos de malária diretamente associados ao avanço do desmatamento^(2)^.

A enfermagem, categoria que representa 56% dos profissionais de saúde na região^(3)^, vivencia essas crises em três dimensões: a) Na atenção à saúde: crianças desidratadas por ondas de calor na Amazônia, idosos com pneumonia agravada pela poluição do ar nas grandes cidades; b) Epidemiológica: vigilância de surtos de leptospirose pós-enchentes e mapeamento de áreas de risco para arboviroses, aumento dos casos de malária, dentre outros; c) Política: falta de saneamento em periferias urbanas, onde internações pediátricas estão ligadas a doenças diarreicas de origem hídrica, e falta de política para conter a poluição do ar, responsável por mortes prematuras anuais na região, sobrecarregando sistemas de saúde já frágeis.

A escolha da Amazônia como sede da COP30 não é casual, mas antes reflete tanto a crise iminente de um bioma essencial à regulação do clima global quanto a resistência histórica dos povos tradicionais que, por meio de saberes ancestrais, asseguram a conservação de ecossistemas críticos. Para o campo da enfermagem, essa conferência impõe três imperativos fundamentais:

1. Financiamento equitativo

Apesar de as questões climáticas afetarem diretamente a saúde das populações, apenas 0,5% dos recursos financeiros globais para mitigação e adaptação ao clima são alocados ao setor saúde. Entretanto, o impacto econômico das enfermidades associadas às alterações ambientais na América Latina supera anualmente 2 bilhões de dólares^(4)^. Torna-se, portanto, imperativo redirecionar verbas para:

• A implementação de infraestrutura hospitalar resiliente a desastres naturais.

• A formação continuada em saúde ambiental para profissionais de enfermagem da região, com metas claras até 2030.

2. Ciência integrada aos saberes tradicionais

Protocolos convencionais muitas vezes revelam-se ineficazes em contextos ribeirinhos ou de práticas indígenas de manejo do meio - como sistemas agroflorestais - que demonstram redução de até 40% na incidência de enfermidades transmitidas por vetores^(2)^. Nesse sentido, a enfermagem detém um papel estratégico como mediadora entre o conhecimento científico ocidental e os saberes de povos tradicionais, contribuindo para a formulação de políticas públicas culturalmente adequadas e ambientalmente sustentáveis.

3. Sistemas de saúde sustentáveis e resilientes

O próprio setor de saúde é responsável por cerca de 4,4% das emissões globais de CO₂^(5)^. A adoção de fontes renováveis, como energia solar em estabelecimentos hospitalares, aliada a políticas de redução de resíduos plásticos, configura-se como medida viável e urgente. Várias instituições no mundo, já conseguiram demostrar avanços da assistência à saúde de baixo carbono, infelizmente, poucas na América Latina. Essas iniciativas não apenas promovem a sustentabilidade ambiental, mas também fortalecem a resiliência institucional frente aos desafios climáticos^(6)^.

A COP30 não será apenas mais uma conferência. É uma oportunidade para a enfermagem latino-americana liderar um movimento que una ciência, política e justiça social. Passos fundamentais para essa estratégia incluem, mas não se limitam a:

• Incluir a crise climática nos currículos de graduação e pós-graduação, formando profissionais aptos a atuar em cenários de incerteza ambiental.

• Fortalecer redes de pesquisa interdisciplinares, documentando os impactos do clima na saúde materno-infantil, mental e saúde do trabalhador.

• Ocupar espaços de decisão, dos conselhos municipais às delegações oficiais na COP30, defendendo que não há neutralidade possível diante da devastação.

A crise climática exige que a enfermagem transcenda seu papel tradicional e assuma liderança na defesa de um futuro onde cuidar das pessoas e do planeta sejam faces da mesma moeda.

A história nos julgará pela coragem de ir além dos sintomas e enfrentar as feridas abertas da injustiça que atravessam corpos e territórios. A inércia não é opção. Que a enfermagem latino-americana, forjada na luta e na resistência, se levante mais uma vez para dizer ao mundo: não há cuidado verdadeiro sem justiça, nem futuro possível sem compromisso com a saúde planetária.

# Translation: Enfermería y la crisis climática - El llamado de la COP30 para la salud en América Latina

**DOI:** 10.1590/1518-8345.0000.4679

**Authors:** Ethel Leonor Maciel

**Affiliations:** 1 Universidade Federal do Espírito Santo, Vitória, ES, Brasil.



La emergencia climática es una de las mayores amenazas para la salud global del siglo XXI. La declaración aprobada durante la reunión ministerial del G20 en Brasil, titulada “Declaración Ministerial sobre Cambio Climático, Salud, Equidad y Una Sola Salud”, consolida este hito al reconocer la crisis climática como una amenaza directa a la salud global, especialmente para poblaciones vulnerables. El documento refuerza la necesidad de integrar políticas climáticas y sanitarias para combatir enfermedades zoonóticas y los efectos de eventos extremos como olas de calor, inundaciones y sequías. Los líderes del G20 también se comprometieron a priorizar la adaptación de los sistemas de salud, garantizando acceso equitativo a recursos y tecnologías, y a ampliar el financiamiento para proyectos que reduzcan emisiones de gases de efecto invernadero en el sector salud^(1)^.

En América Latina, donde los efectos del calentamiento global se entrelazan con desigualdades históricas, la enfermería emerge no solo como testigo, sino como protagonista en la construcción de respuestas éticas y efectivas. Mientras los líderes globales se preparan para la 30ª Conferencia de las Partes de la ONU sobre Cambio Climático (COP30), que se celebrará en noviembre de 2025 en Belém, Pará, Brasil, urge reflexionar: ¿Cómo transformar el cuidado humano en acción colectiva por el planeta?

La región presenta una paradoja marcada: a pesar de albergar cerca del 40% de la biodiversidad global, figura entre las más vulnerables a los efectos climáticos. En los últimos diez años, Brasil registró un aumento del 35% en inundaciones, mientras que la incidencia de enfermedades tropicales como dengue y chikungunya se duplicó. Además, las comunidades indígenas - responsables de conservar el 80% de los bosques remanentes - enfrentan altas tasas de desnutrición infantil y brotes de malaria vinculados a la deforestación^(2)^.

La enfermería, que representa el 56% de los profesionales de salud en la región^(3)^, vive estas crisis en tres dimensiones: a) Atención sanitaria: niños deshidratados por olas de calor en la Amazonía, ancianos con neumonía agravada por contaminación urbana; b) Epidemiológica: vigilancia de leptospirosis post-inundaciones, mapeo de zonas de riesgo para arbovirosis y aumento de malaria; c) Política: falta de saneamiento en periferias urbanas - con hospitalizaciones pediátricas por enfermedades hídricas - y ausencia de políticas contra la contaminación del aire, que causa muertes prematuras y satura sistemas de salud frágiles.

La elección de la Amazonía como sede de la COP30 no es casual. Refleja tanto la crisis de un bioma esencial para el clima global como la resistencia histórica de pueblos tradicionales, cuyos saberes ancestrales preservan ecosistemas críticos. Para la enfermería, esta conferencia impone tres imperativos:

1. Financiamiento equitativo

Aunque el clima impacta directamente la salud, solo el 0,5% de los fondos globales para mitigación y adaptación climática se destina al sector salud. Sin embargo, el costo económico de las enfermedades vinculadas al ambiente en América Latina supera los 2 mil millones de dólares anuales^(4)^. Es crucial redirigir recursos hacia:

• Infraestructura hospitalaria resistente a desastres.

• Capacitación en salud ambiental para enfermeros, con metas claras al 2030.

2. Ciencia integrada a saberes tradicionales

Los protocolos convencionales suelen ser ineficaces en contextos ribereños o indígenas - como sistemas agroforestales, que reducen un 40% las enfermedades vectoriales^(2)^. La enfermería debe mediar entre el conocimiento científico y los saberes tradicionales para políticas públicas culturalmente adecuadas y sostenibles.

3. Sistemas de salud sostenibles y resilientes

El sector salud contribuye el 4,4% de las emisiones globales de CO₂^(5)^. La adopción de energías renovables (ej. solar en hospitales) y la reducción de residuos plásticos son medidas urgentes. Aunque existen ejemplos globales, América Latina avanza lentamente. Estas iniciativas fortalecen la sostenibilidad y la resiliencia institucional^(6)^.

La COP30 no será otra conferencia más. Es una oportunidad para que la enfermería latinoamericana lidere un movimiento que una ciencia, política y justicia social. Pasos clave incluyen:

• Incorporar la crisis climática en los currículos de enfermería, formando profesionales para escenarios de incertidumbre ambiental.

• Fortalecer redes de investigación interdisciplinaria sobre impactos climáticos en salud materno-infantil, mental y laboral.

• Ocupar espacios de decisión - desde consejos municipales hasta delegaciones en la COP30 - rechazando la neutralidad ante la devastación.

La crisis climática exige que la enfermería trascienda su rol tradicional y lidere un futuro donde cuidar de las personas y el planeta sean una misma lucha. La historia nos juzgará por el coraje de enfrentar las injusticias que marcan cuerpos y territorios. La inercia no es opción. Forjada en la lucha, la enfermería latinoamericana debe alzar su voz nuevamente: no hay cuidado verdadero sin justicia, ni futuro posible sin salud planetaria.
